# Femoroacetabular Impingement Randomised controlled Trial (FIRST) - a multi-centre randomized controlled trial comparing arthroscopic lavage and arthroscopic osteochondroplasty on patient important outcomes and quality of life in the treatment of young adult (18–50 years) femoroacetabular impingement: a statistical analysis plan

**DOI:** 10.1186/s13063-018-2965-0

**Published:** 2018-10-29

**Authors:** Nicole Simunovic, D. Heels-Ansdell, L. Thabane, O. R. Ayeni, on behalf of the FIRST Investigators, Olufemi R. Ayeni, Mohit Bhandari, Asheesh Bedi, Teppo Järvinen, Volker Musahl, Douglas Naudie, Matti Seppänen, Gerard Slobogean, Lehana Thabane, Nicole Simunovic, Andrew Duong, Matthew Skelly, Sheila Sprague, Diane Heels-Ansdell, Lisa Buckingham, Tim Ramsay, John Lee, Petteri Kousa, Sasha Carsen, Hema Choudur, Yan Sim, Kelly Johnston, Ivan Wong, Nicole Paquet, Jalisa Den Hartog, Daniel Whelan, Ryan Khan, Gavin C. A. Wood, Fiona Howells, Heather Grant, Bryn Zomar, Michael Pollock, Kevin Willits, Andrew Firth, Stacey Wanlin, Alliya Remtulla, Nicole Kaniki, Etienne L. Belzile, Sylvie Turmel, Mari Pirjetta Routapohja, Uffe Jørgensen, Annie Gam-Pedersen, Raine Sihvoenen, Marko Raivio, Pirjo Toivonen

**Affiliations:** 1 Department of Health Research Methods, Evidence and Impact, McMaster University, 1200 Main St W, 2C, Hamilton, ON L8S 4K1 Canada; 2 Department of Surgery, Division of Orthopaedic Surgery, McMaster University, 1200 Main St W, 4E15, Hamilton, ON L8N 3Z5 Canada; 3 Biostatistics/FORSC, 3rd Floor, H325, St. Joseph’s Healthcare, 50 Charlton Ave. E, Hamilton, ON L8N 4A6 Canada

**Keywords:** Statistical analysis plan, Randomised controlled trial, Femoroacetabular impingement, Lavage, Osteochondroplasty

## Abstract

**Background:**

The research objectives of the Femoroacetabular Impingement Randomised controlled Trial (FIRST) are to assess whether surgical correction of the hip impingement morphology (arthroscopic osteochondroplasty) with or without labral repair, in adults aged 18–50 years diagnosed with non-arthritic femoroacetabular impingement (FAI), provides decreased pain and improved health-related quality of life at 12 months compared to arthroscopic lavage of the hip joint. This article describes the statistical analysis plan for the FIRST trial.

**Methods/design:**

FIRST is an ongoing multi-centre, blinded randomised controlled trial of 220 patients who have been diagnosed with FAI and are optimized for surgical intervention. This article describes the overall analysis principles, including how participants will be included in each analysis, the presentation of the results, adjustments for covariates, the primary and secondary outcomes and their respective analyses. In addition, we will present the planned sensitivity and subgroup analyses.

**Discussion:**

Our rationale for FIRST is based upon (1) an epidemic of FAI surgery with resultant increased healthcare costs over that last decade, (2) worldwide disparity in perceptions about its utility, and (3) consensus that definitive evidence for or against surgical approaches is lacking.

**Trial registration:**

ClinicalTrials.gov, NCT01623843. Registered on 20 June 2012.

## Background

The Femoroacetabular Impingement Randomised controlled Trial (FIRST) is a multi-centre, concealed randomized controlled trial (RCT) evaluating the effect of arthroscopic lavage (i.e. washing out the hip joint) versus osteochondroplasty (i.e. surgical correction of the hip impingement morphology) in adults aged 18–50 years diagnosed with non-arthritic femoroacetabular impingement (FAI). The protocol for the FIRST trial has been previously published [1] and provides more detail on the trial rationale, eligibility criteria, interventions, data management, and methods for limiting bias.

FAI is a condition that causes hip pain in the young adult as a result of a size and shape mismatch between the femoral head and the acetabulum. FAI is typically classified into two sub-types; cam type (a misshaped femoral head) or pincer type (an over-covered or deep socket). Most patients have a combination of both types of impingement. With FAI, the femoral head (ball) and acetabular rim (socket) of the hip joint collide during hip flexion and rotation. This collision results in an impingement of the femoral head/neck/column on the acetabular rim, and patients experience hip pain. This pain can be a precursor to early hip damage such as cartilage delamination and labral tears of the hip. As the condition progresses, the resulting hip damage may lead to osteoarthritis of the hip [1].

The rationale for the FIRST trial is based upon (1) an exponential increase in FAI surgery with resultant increased healthcare costs over that last decade, (2) worldwide disparity in perceptions about its utility, and (3) consensus that definitive evidence for or against surgical approaches is lacking. Therefore, the primary objective of the trial is to assess whether surgical correction of the impingement morphology (arthroscopic osteochondroplasty) with or without labral repair, in adults aged 18–50 years diagnosed with non-arthritic FAI, provides decreased pain at 12 months compared to arthroscopic lavage of the hip joint. Secondary objectives include measuring outcomes associated with the intervention and control groups (osteochondroplasty versus lavage) related to improved lifestyle, emotional health, and physical health.

**Fig. 1:**
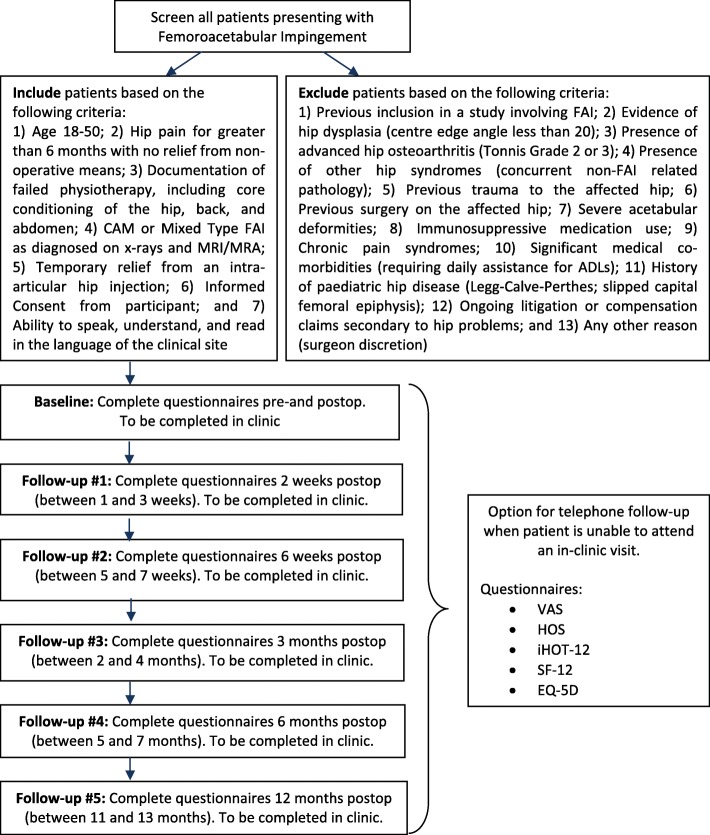
Femoroacetabular Impingement Randomised controlled Trial (FIRST) process overview. MRI, magnetic resonance imaging; MRA, magnetic resonance angiography; FAI, femoroacetabular impingement; VAS, visual analogue scale; HOS, Hip Outcome Score; iHOT, International Hip Outcome Tool; SF-12 Short Form-12; EQ-5D, EuroQol-5 Dimensions

This trial is a parallel multi-centre, blinded randomised controlled trial (RCT) of 220 patients who have been diagnosed with FAI, to determine the superiority of arthroscopic osteochondroplasty to arthroscopic lavage. Briefly, participants were recruited from experienced hip surgeons practicing at 10 participating sites based in Canada, Finland, and Denmark. Patients were allocated to one of two treatment arms using an online centralized 24-h computerised randomisation system. The randomisation system follows a computer-generated randomisation schedule in random block sizes of 4 and 8. Randomisation was stratified by impingement sub-type (cam versus mixed) and clinical centre. Study personnel monitor critical aspects of perioperative care and rehabilitation. We are assessing subject pain within 12 months using a visual analogue scale (VAS) after surgery as the primary outcome. Secondary outcomes include function, health-related quality of life, post-operative complications, and costs. Quality of the surgery and complications, including re-operations, will be reviewed by an independent adjudication committee. Outcome assessors and data analysts are blinded to treatment allocation. The full study process is shown in Fig. 1.

In this article, we present our planned statistical analyses for the FIRST trial. The statistical analysis plan was finalized and approved on 29 November 2017 (Version 1.0) for the FIRST trial protocol (20 April 2016, Version 3.0) and in accordance with the trial Masterfile, including the Data Management Plan (June 4, 2014, Version 1.0). Ethics approval was granted at the Methods Centre at McMaster University (Hamilton Integrated Research Ethics Board #12–396) and at each participating site (as per their local ethics board). The trial is registered at ClinicalTrials.gov (NCT01623843).

## Methods

### Outcomes

#### Primary outcome

The FIRST primary outcome is pain at 12 months as measured by the VAS. The primary analysis is to assess whether surgical correction of the impingement morphology (arthroscopic osteochondroplasty) with/without labral repair, in adults aged 18–50 years diagnosed with FAI, provides decreased pain at 12 months compared to arthroscopic lavage of the hip joint with/without labral repair, as measured by the VAS. The VAS is a validated unidimensional scale that is easy to use, requires no verbal or reading skills, and is sufficiently versatile to be employed in a variety of settings [2–4].

#### Secondary outcomes

Secondary outcomes include:

1. Hip function as measured by the Hip Outcome Score (HOS).
2. Generic physical and mental health as measured by the Short Form-12 (SF-12).
3. Impact of hip-specific disease on function and lifestyle in the young, active patient as measured by the International Hip Outcome Tool (iHOT-12).
4. Health utility as measured by the EuroQol (EQ-5D).
5. Complications, including additional surgery and other serious and non-serious adverse events. Reasons for re-operations for the randomized hip typically include, but are not limited to re-injury of the labrum/cartilage, hip dislocation, hip instability, infection (deep or superficial), wound healing problem, soft tissue problem, and unresolved hip pain. Other hip-related adverse events to be reported include, but are not limited to, hip instability, tendinopathy, re-injury of the labrum/cartilage, hip osteoarthritis post-surgery, and infection (superficial or deep).

The HOS is a self-administered hip score that was designed to capture hip function and outcomes following surgical therapies such as arthroscopy [5]. The HOS has been shown to have the greatest clinimetric evidence for use in patients with FAI or labral tears [6, 7]. The SF-12 may be self-completed or interview-administered and will help document general health status and the burden of illness that FAI presents [8]. The iHOT-12 is a shorter version of the iHOT-33 designed to be easier to complete in routine clinical practice to measure both health-related quality of life and changes after treatment in young, active patients with hip disorders [9]. This questionnaire has been shown to be valid, reliable, and responsive to change [9]. The EQ-5D is a standardized instrument for use as a measure of health outcome [10]. The EQ-5D comprises five dimensions of health (mobility, self-care, usual activities, pain/discomfort, and anxiety/depression). The EQ-5D has been used in previous studies involving patients with hip pain and has been extensively validated [11, 12].

## Discussion

### Analysis plan

This statistical analysis plan follows the JAMA Guidelines for the content of statistical analysis plans in clinical trials [13]. A summary of all planned analyses is provided in Table 1.

**Table 1:** Statistical analysis plan summary

Objective	Outcome		Hypothesis	Method of analysis^a^
	Name	Type		
Primary objective				
To compare pain levels at 1 year	Pain (VAS)	Continuous	Osteochondroplasty will reduce pain compared to lavage	Multiple linear regression
Secondary objectives 1				
To compare patient-reported health-related quality of life	Hip function (HOS)	Continuous	Osteochondroplasty will improve health-related quality of life, function, and utility compared to lavage	Multiple linear regression
	Hip-specific disease on hip function (iHOT-12)	Continuous		
	Physical health (SF-12 PCS)	Continuous		
	Mental health (SF-12 MCS)	Continuous		
	Health Utility (EQ-5D)	Continuous		
Secondary objective 2				
To compare hip complications	Hip-related complications (e.g. re-operation)	Binary	Osteochondroplasty will reduce rate of re-operations compared to lavage	Multiple logistic regression
Subgroup analysis				
Hip impingement severity: mild (alpha angle < 60 – > 50 degrees), moderate (alpha angle > 60 – < 83°), severe (alpha angle > 83°)	Pain (VAS)	Continuous	Patients with severe impingement at baseline will have the greatest improvement with the osteochondroplasty procedure compared with those with moderate to mild impingement	Multiple linear regression
Gender: male, female	Pain (VAS)	Continuous	The osteochondroplasty procedure will perform better in males	Multiple linear regression
Cartilage status (based on Tonnis and Heinecke classification): grades 3 and 4, grades 1 and 2	Pain (VAS)	Continuous	Osteochondroplasty will perform worse in patients with worse cartilage status (i.e. grades 3 and 4)	Multiple linear regression
Treatment of the labrum: labral repair, resection	Pain (VAS)	Continuous	Patients receiving a labral repair will perform better than those receiving a resection as part of the osteochondroplasty procedure	Multiple linear regression
Sensitivity analysis				
Trial site (centre-effects)	Pain (VAS)	Continuous	We do not expect the effect to change substantially when centre-effects are removed from the primary analysis	Multiple linear regression with centre-effects removed
Missing data effect	Pain (VAS)	Continuous	We do not expect the effect to change substantially without imputation for missing data	Multiple linear regression with complete cases only
Potential baseline imbalance	Pain (VAS)	Continuous	Results will remain robust after adjusting for potential baseline imbalance on age, any comorbidities, onset of symptoms, and presence of labral tears at initial surgery	Multiple linear regression with complete cases only

*All regression analyses will be controlled for centre as a stratification variableVAS: Visual Analogue Scale, SF: Short Form, PCS: Physical Component Summary, MCS: Mental Component Summary, HOS:Hip Outcome Score, iHOT: International Hip Outcome Tool, EQ-5D: Euroqol-5 Dimensions

#### Blinded analyses

All statistical analyses will first be completed using blinded treatment groups (i.e. treatment X and Y). Interpretations for the effect of the surgical interventions will be documented based upon blinded X versus Y treatment [14].

#### Presentation of data

The trial results will be presented according to the Consolidated standards of reporting trials (CONSORT) guidelines for RCTs [15]. The baseline demographic characteristics and a description of the surgical and peri-operative management characteristics of the patients will be summarized by group, reported as mean (standard deviation (SD)) or median (first quartile, third quartile) for continuous variables and count (percent) for categorical variables (Tables 2 and 3). All statistical tests will be two-tailed with α = 0.05.

**Table 2:** Patient demographics and hip characteristics

	Treatment X *n* =	Treatment Y *n* =
Patient characteristics		
Age, mean (SD)		
Gender, *n* (%)		
Male		
Female		
Ethnicity, *n* (%)		
Native		
Asian		
Black		
Hispanic		
White/Caucasian		
Smoking history, *n* (%)		
Never smoked		
Current smoker		
Former smoker		
Alcohol consumption, *n* (%)		
No alcohol at baseline		
0.5–2 drinks/week		
3–5 drinks/week		
6–10 drinks/week		
11+ drinks/week		
Current medications, *n* (%)		
None		
NSAIDS		
Intra-articular injection		
Etc…. (as per available data)		
Co-morbidities, *n* (%)		
None		
Cancer		
Back pain		
Etc… (as per available data)		
BMI, *n* (%)		
Underweight < 18.5		
Normal weight 18.5–24.9		
Overweight 25–29.9		
Obese 30–39.9		
Morbidly obese 40 or greater		
Weightbearing status, *n* (%)		
Full weightbearing		
Partial weightbearing		
Non-weightbearing		
Baseline sport activity, *n* (%)		
None		
Light		
Moderate		
Vigorous		
Hip characteristics		
Affected hip, *n* (%)		
Left		
Right		
Location of hip pain, *n* (%)		
Groin		
Lateral		
Posterior		
Groin and lateral		
Groin and posterior		
Lateral and posterior		
Groin and lateral and posterior		
Onset of symptoms, *n* (%)		
Acute		
Subacute		
Insidious		
Traumatic		
Non-traumatic		
Tonnis and Heinecke classification, *n* (%)		
Grade 0		
Grade 1		
Grade 2		
Grade 3		
Labral tears present, *n* (%)		
None		
Anterior		
Posterior		
Superior/lateral		
Anterior and posterior		
Anterior and superior/lateral		
Posterior and superior/lateral		
Herniation pits present, *n* (%)		
No		
Yes		

*NSAID* non-steroidal anti-inflammatory drug, *BMI* body mass index

**Table 3:** Surgical and peri-operative management

	Treatment X *n* =	Treatment Y *n* =
Duration of procedure, mean (SD)		
Duration of traction, mean (SD)		
Total saline used in procedure, mean (SD)		
Type of surgical prep solution, *n* (%)		
Iodine		
Chlorohexidine		
Alcohol		
Etc... (as per available data)		
Labral tears, *n* (%)		
None		
Partial		
Complete		
Labrum injected, *n* (%)		
No		
Yes		
Focal		
Diffuse		
Outerbridge intra-operative cartilage classification, *n* (%)		
Grade 0		
Grade 1		
Grade 2		
Grade 3		
Grade 4		
Beck intra-operative cartilage classification, *n* (%)		
Grade 0		
Grade 1		
Grade 2		
Grade 3		
Grade 4		
Beck intra-operative labral classification, *n* (%)		
Grade 0		
Grade 1		
Grade 2		
Grade 3		
Grade 4		
Capsulotomy performed, *n* (%)		
No		
Yes		
Partial		
Complete		
Capsular closure performed, *n* (%)		
Yes		
No		
Anchors used for labrum repair, *n* (%)		
Not applicable (no repair)		
0		
1		
2		
3		
4		
5		
6		
Antibiotic prophylaxis, *n* (%)		
No		
Yes		
Cefazolin		
Cefuroxime		
Vancomycin		
Other		
Thromboprophylaxis, *n* (%)		
No		
Yes		
Aspirin		
Heparin		
Warfarin		
Mechanical		
LMWH		
Other		
Patient discharge location, *n* (%)		
Home		
Rehabilitation facility		
Other hospital		
Weightbearing, *n* (%)		
Non-weightbearing		
Partial weightbearing		
Full weightbearing		
Patient aids at discharge, *n* (%)		
None (ambulatory)		
Wheelchair		
Walker		
Two crutches		
One crutch		
Cane		
Other		

*LMWH* low molecular-weight heparin

#### Primary outcome analysis

Our hypotheses for the primary analysis are as follows:

- Null hypothesis: there is no difference in reported pain between groups at 12 months measured using the VAS score.
- Alternative hypothesis: there is a difference in reported pain between groups at 12 months measured using the VAS score.

The primary analysis will be an analysis to compare the mean pain scores (VAS) at 12 months post-surgery adjusting for baseline VAS score (Table 4). This analysis will be a multiple linear regression with VAS as the dependent variable and the following independent variables: treatment, baseline VAS score, impingement subtype, and clinical centre (all centres with fewer than 10 patients enrolled will be collapsed into a single centre for the independent variable entered into the primary analysis model). Assuming that data would be missing at random, we will use multiple imputation that will be stratified by trial arm and will include baseline demographic or prognostic variables for which we have complete data to handle missing data to enable intention-to-treat analysis [16]. The treatment effect will be reported as an absolute difference in rate of pain reduction with the associated 95% confidence interval and *p* value. We will not perform a per-protocol analysis given the cross-over rate at the time of final enrollment was less than 0.5%. We will not exclude cross-overs in the final analysis. We will examine residuals to assess the model assumptions for the multiple linear regression model. All analyses will be performed using SAS version 9.4 (Cary, NC, USA).

**Table 4:** Study outcomes by treatment group

	Treatment X *n* =	Treatment Y *n* =	Mean difference^a^ (95% CI)	*p* value
	mean (SD)	mean (SD)		
Primary outcome (pain as measured by VAS)				
Secondary outcomes				
SF-12 PCS				
SF-12 MCS				
HOS				
iHOT-12				
EQ-5D utility score				
	*n* (%)	*n* (%)	Odds ratio^b^ (95% CI)	*p* value
Hip-related complications				
Re-operations				

*VAS* visual analogue scale, *PCS* physical component summary, *MCS* mental component summary, *HOS* Hip Outcome Score, *iHOT* International Hip Outcome Tool, *EQ-5D* Euroqol-5 Dimensions, SD standard deviation, CI confidence interval

^a^From the multiple linear regression model

^b^From the logistic regression model

#### Secondary outcomes analysis

We will estimate the effect of arthroscopic osteochondroplasty (intervention) versus lavage (control) on FAI patient quality of life (SF-12 mental component summary (MCS) and physical component summary (PCS)), function HOS, iHOT-12), and health utility (EQ-5D) at 12 months (Table 4). Similar to the primary analysis, we will perform multiple linear regressions that include treatment, baseline score, impingement subtype, and centre as independent variables. The results will be reported as mean differences with 95% confidence intervals. We will also estimate the effect of arthroscopic osteochondroplasty (intervention) versus lavage (control) on re-operation using logistic regression that includes treatment and impingement sub-type as independent variables. If we observe enough events, we will also include centre as an independent variable. The results will be presented as the odds ratio (OR) with the 95% confidence interval. Other hip-related adverse events that were not treated operatively will be presented by randomised group. The *p* values for treatment effects for these outcomes will not be adjusted given that the secondary analyses will be exploratory. We will also report hip measurements pre-surgery/post-surgery and 12 months post-surgery by treatment group (Table 5). Analyses for secondary outcomes will be complete-case analyses only.

**Table 5:** Hip measurements

	Treatment X			Treatment Y		
	*n* =	*n* =	*n* =	*n* =	*n* =	*n* =
	Pre-op	Post-op	12 Months	Pre-op	Post-op	12 Months
Anterior hip impingement test, *n* (%)						
Positive						
Negative						
Posterior hip impingement test, *n* (%)						
Positive						
Negative						
Log roll test, *n* (%)						
Positive						
Negative						
Crossover sign, *n* (%)						
Positive						
Negative						
Coxa profunda, *n* (%)						
Positive						
Negative						
Coxa protrusio, *n* (%)						
Positive						
Negative						
Centre-edge angle, mean (SD)						
Alpha angle, mean (SD)						
Neck shaft angle, mean (SD)						
Femoral offset ratio, mean (SD)						
Study hip range of motion, mean (SD)						
Flexion						
Extension						
Abduction						
Adduction						
Internal rotation (neutral)						
External rotation (neutral)						
Internal rotation (90° flexion)						
External rotation (90° flexion)						
Non-study hip range of motion, mean (SD)						
Flexion						
Extension						
Abduction						
Adduction						
Internal rotation (neutral)						
External rotation (neutral)						
Internal rotation (90° flexion)						
External rotation (90° flexion)						

*Pre-op* preoperative

### Sensitivity analyses

We will perform sensitivity analysis of centre-effects*,* where we will repeat the primary analysis where clinical centre is not included in the model. We will also perform sensitivity analysis in regards to missing data where we include only complete cases (i.e. no imputation for missing data) [17, 18]. We will also conduct an adjusted analysis, which will adjust for baseline demographics, which we reasonably expect to have an impact on our trial outcomes. We will add the following to the primary analysis as independent variables: (1) age (under 40 years vs. 40 years and older), (2) any comorbidities reported at baseline, (3) onset of symptoms (acute, subacute, insidious, traumatic, non-traumatic), and (4) presence of labral tears at initial surgery. This will address any potential baseline imbalance between randomised groups.

### Subgroup analyses

**Fig. 2:**
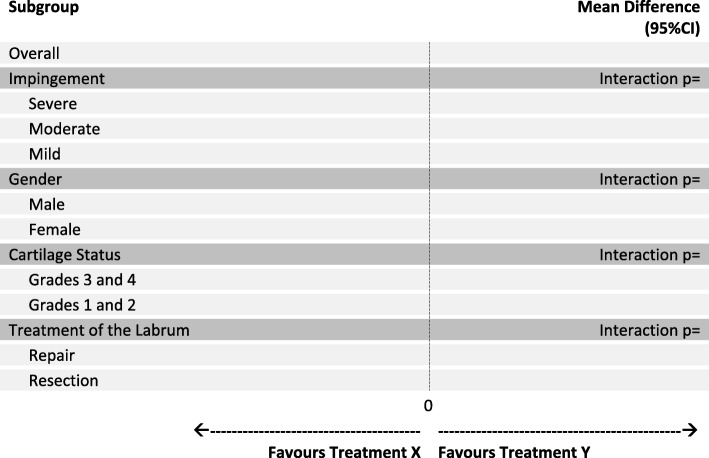
Subgroup analyses of the primary end point, according to treatment group

At the onset of the FIRST trial, we identified 4 important subgroups, which will be reported according to standard guidelines [19]. We will add a main effect for the subgroup variable and the treatment by subgroup interaction to our primary model described above to assess whether the magnitude of the treatment effect is significantly different between subgroups (Fig. 2). This will be repeated for each subgroup variable. We will perform these subgroup analyses with the primary endpoint as the outcome:

1. Severe versus moderate versus mild baseline impingement - impingement will be classified as follows: severe (alpha angle greater than 83°), moderate (alpha greater than 60°), and mild (alpha angle of less than 60°). We hypothesize that patients with severe impingement at baseline will have the greatest improvement with the osteochondroplasty procedure compared with those with moderate to mild impingement [20–22].
2. Gender **-** we hypothesize that the osteochondroplasty procedure will perform better in men [23, 24].
3. Cartilage status (grades 3 and 4 vs 1 and 2) - based on the Tonnis and Heinecke cartilage classification, we hypothesize that osteochondroplasty will perform worse in patients with worse cartilage status (i.e. grades 3 and 4) [25–27].
4. Treatment of the labrum **-** we hypothesize that patients receiving a labral repair will perform better than those receiving a resection as part of the osteochondroplasty procedure [28].

If a statistically significant subgroup effect is found, we will further explore the impact of the subgroup on the secondary outcomes. No interim analyses are planned due to our desire to avoid spuriously inflated estimates of treatment effect [29, 30]. The Data Safety and Monitoring Committee (DSMC) meet regularly to monitor the study data for safety.

#### Dissemination

Upon trial completion, the primary manuscript with the 12-month follow-up results, whether positive, negative or neutral, will be submitted for peer-reviewed publication in a top medical journal. The final dataset will be shared through an open access data repository once all analyses are completed.

### Trial status

The trial began as a pilot of 50 patients in November 2012. Upon demonstrating feasibility and securing additional funding (January 2015), these participants were rolled into the definitive trial (*N* = 220). For the definitive trial, full participant recruitment was achieved in November 2017 and the final 12-month follow-up is expected to be completed in December 2018.

## Abbreviations

EQ-5D: EuroQol-5 Dimensions
FAI: Femoroacetabular impingement
FIRST: Femoroacetabular impingement randomised controlled trial
HOS: Hip Outcome Score
iHOT-12: International Hip Outcome Tool-12
MCS: Mental component summary
OR: Odds ratio
PCS: Physical component summary
RCT: Randomised controlled trial
SD: Standard deviation
SF-12: Short Form-12
VAS: Visual analogue scale

## References

[1] FIRST Investigators (2015). A multi-centre randomized controlled trial comparing arthroscopic osteochondroplasty and lavage with arthroscopic lavage alone on patient important outcomes and quality of life in the treatment of young adult (18-50) femoroacetabular impingement. BMC Musculoskelet Disord.

[2] Jensen MP, Karoly P (1986). The measurement of clinical pain intensity: a comparison of six methods. Pain.

[3] Collins S, Moore A, McQuay H (1997). The visual analog pain intensity scale: what is moderate pain in millimeters?. Pain.

[4] Ho K, Spence J, Murphy M (1996). Review of pain measurement tools. Ann Emerg Med.

[5] Schenker ML, Martin R, Weiland DE, Philippon MJ (2005). Current trends in hip arthroscopy: a review of injury diagnosis, techniques and outcome scoring. Curr Opin Orthopeadics.

[6] Thorborg K, Roos EM, Bartels EM, Petersen J, Hölmich P (2010). Validity, reliability and responsiveness of patient-reported outcome questionnaires when assessing hip and groin disability: a systematic review. Br J Sports Med.

[7] Mohtadi NGH, Griffin DR, Pedersen ME, Chan D, Safran MR, Parsons N, Sekiya JK, Kelly BT, Werle JR, Leunig M, JC MC, Martin HD, Byrd T, Philippon MJ, Martin RL, Guanche CA, Clohisy JC, Sampson TG, Kocher MS, Larson CM, for the Multicenter Arthroscopy of the Hip Outcomes Research Network (MAHORN) (2012). The development and validation of a self-administered quality-of-life outcome measure for young, active patients with symptomatic hip disease: the International Hip Outcome Tool (iHOT-33). Arthroscopy.

[8] Ware J, Kosinski M, Keller SD (1996). A 12-item short-form health survey: construction of scales and preliminary tests of reliability and validity. Med Care.

[9] Griffin DR, Parsons N, Mohtadi NGH, Safran MR, on behalf of the Multicenter Arthroscopy of the Hip Outcomes Research Network (MAHORN) (2012). A short version of the International Hip Outcome Tool (iHOT-12) for use in routine clinical practice. Arthroscopy.

[10] EuroQol Group. The EQ-5D. Available at: http://www.euroqol.org/home.html. Accessed 10 Jan 2018.

[11] Bosch JL, Hunink MG (2000). Comparison of the Health Utilities Index Mark 3 (HUI3) and the EuroQol EQ-5D in patients treated for intermittent claudication. Qual Life Res.

[12] Hurst NP, Kind P, Ruta D, Hunter M, Stubbings A (1997). Measuring health-related quality of life in rheumatoid arthritis: validity, responsiveness and reliability of EuroQol (EQ-5D). Br J Rheumatol.

[13] Gamble C, Krishan A, Stocken D, Lewis S, Juszczak E, Dore C, Williamson PR, Altman DG, Montgomery A, Lim P, Berlin J, Senn S, Day S, Barbachano Y, Loder E (2017). Guidelines for the content of statistical analysis plans in clinical trials. JAMA.

[14] Jarvinen TL, Sihvonen R, Bhandari M, Sprague S, Malmivaara A, Paavola M, Schunemann HJ, Guyatt GH (2014). Blinded interpretation of study results can feasibly and effectively diminish interpretation bias. J Clin Epidemiol.

[15] Altman DG, Moher D, Schulz KF, for the CONSORT Group (2010). CONSORT 2010 statement: updated guidelines for reporting parallel group randomised trials. BMJ.

[16] Zhang Y, Alyass A, Vanniyasingam T, Sadeghirad B, Florez ID, Pichika SC, Kennedy SA, Abdulkarimova U, Zhang Y, Iljon T, Morgano GP, Colunga Lozano LE, Aloweni FAB, Lopes LC, Yepes-Nunez JJ, Fei Y, Wang L, Kahale LA, Meyre D, Akl EA, Thabane L, Guyatt GH (2017). A systematic survey of the methods literature on the reporting quality and optimal methods of handling participants with missing outcome data for continuous outcomes in randomized controlled trials. J Clin Epidemiol.

[17] Chu R, Thabane L, Ma J, Holbrook A, Pullenayegum E, Devereaux PJ (2011). Comparing methods to estimate treatment effects on a continuous outcome in multicentre randomized controlled trials: a simulation study. BMC Med Res Methodol.

[18] Thabane L, Mbuagbaw L, Zhang S, Samaan Z, Marcucci M, Ye C, Thabane M, Giangregorio L, Dennis B, Kosa D, Borg Debono V, Dillenburg R, Fruci V, Bawor M, Lee J, Wells G, Goldsmith CH (2013). A tutorial on sensitivity analyses in clinical trials: the what, why, when and how. BMC Med Res Methodol.

[19] Wang R, Lagakos SW, Ware JH, Hunter DJ, Drazen JM (2007). Statistics in medicine – reporting of subgroup analyses in clinical trials. N Engl J Med.

[20] Agricola R, Heijboer MP, Bierma-Zeinstra SM, Verhaar JA, Weinans H, Waarsing JH (2013). Cam impingement causes osteoarthritis of the hip: a nationwide prospective cohort study (CHECK). Ann Rheum Dis.

[21] Agricola R, Waarsing JH, Thomas GE, Carr AJ, Reijman M, Bierma-Zeinstra SM, Glyn-Jones S, Weinans H, Arden NK (2014). Cam impingement: defining the presence of a cam deformity by the alpha angle: data from the CHECK cohort and Chingford cohort. Osteoarthr Cartil.

[22] Agricola R, Waarsing JH, Arden NK, Carr AJ, Bierma-Zeinstra SM, Thomas GE, Weinans H, Glyn-Jones S (2013). Cam impingement of the hip-a risk factor for hip osteoarthritis. Nat Rev Rheumatol.

[23] Thomas DD, Bernhardson AS, Bernstein E, Dewing CB (2017). Hip arthroscopy for femoroacetabular impingement in a military population. Am J Sports Med.

[24] Frank RM, Lee S, Bush-Joseph CA, Salata MJ, Mather RC, Nho SJ (2016). Outcomes for hip arthroscopy according to sex and age: a comparative matched-group analysis. J Bone Joint Surg Am.

[25] Tonnis D, Heinecke A (1999). Current concepts review - acetabular and femoral anteversion: relationship with osteoarthritis of the hip. J Bone Joint Surg Am.

[26] Horner NS, Ekhtiari S, Simunovic N, Safran MR, Philippon MJ, Ayeni OR (2017). Hip arthroscopy in patients age 40 or older: a systematic review. Arthroscopy.

[27] Egerton T, Hinman RS, Takla A, Bennell KL, O'Donnell J (2013). Intraoperative cartilage degeneration predicts outcome 12 months after hip arthroscopy. Clin Orthop Relat Res.

[28] Ayeni OR, Adamich J, Farrokhyar F, Simunovic N, Crouch S, Philippon MJ, Bhandari M (2014). Surgical management of labral tears during femoroacetabular impingement surgery: a systematic review. Knee Surg Sports Traumatol Arthrosc.

[29] Briel M, Lane M, Montori VM, Bassler D, Glasziou P, Malaga G, Akl EA, Ferreira-Gonzalez I, Alonso-Coello P, Urrutia G, Kunz R, Culebro CR, da Silva SA, Flynn DN, Elamin MB, Strahm B, Murad MH, Djulbegovic B, Adhikari NKJ, Mills EJ, Gwadry-Sridhar F, Kirpalani H, Soares HP, Elnour NOA, You JJ, Karanicolas PJ, Bucher HC, Lampropulos JF, Nordmann AJ, Burns KEA, Mulla SM, Raatz H, Sood A, Kaur J, Bankhead CR, Mullan RJ, Nerenberg KA, Vandvik PO, Coto-Yglesias F, Schunemann H, Tuche F, Chrispim PPM, Cook DJ, Lutz K, Ribic CM, Vale N, Erwin PJ, Perera R, Zhou Q, Heels-Ansdell D, Ramsay T, Walter SD, Guyatt GH (2009). Stopping randomized clinical trials early for benefit: a protocol of the study of trial policy of interim truncation-2 (STOPIT-2). Trials.

[30] Montori VM, Devereaux PJ, Adhikari NK, Burns KE, Eggert CH, Briel M, Lacchetti C, Leung TW, Darling E, Bryant DM, Bucher HC, Schunemann HJ, Meade MO, Cook DJ, Erwin PJ, Sood A, Sood R, Lo B, Thompson CA, Zhou Q, Mills E, Guyatt GH (2005). Randomized trials stopped early for benefit: a systematic review. JAMA.

